# Synthetic Abortive HIV-1 RNAs Induce Potent Antiviral Immunity

**DOI:** 10.3389/fimmu.2020.00008

**Published:** 2020-01-23

**Authors:** Melissa Stunnenberg, Joris K. Sprokholt, John L. van Hamme, Tanja M. Kaptein, Esther M. Zijlstra-Willems, Sonja I. Gringhuis, Teunis B. H. Geijtenbeek

**Affiliations:** Department of Experimental Immunology, Amsterdam Infection and Immunity Institute, Amsterdam UMC, University of Amsterdam, Amsterdam, Netherlands

**Keywords:** abortive HIV-1 RNA, type I IFN, viral sensing, DDX3, pattern recognition receptor, dendritic cells, antiviral immunity

## Abstract

Strong innate and adaptive immune responses are paramount in combating viral infections. Dendritic cells (DCs) detect viral infections via cytosolic RIG-I like receptors (RLRs) RIG-I and MDA5 leading to MAVS-induced immunity. The DEAD-box RNA helicase DDX3 senses abortive human immunodeficiency virus 1 (HIV-1) transcripts and induces MAVS-dependent type I interferon (IFN) responses, suggesting that abortive HIV-1 RNA transcripts induce antiviral immunity. Little is known about the induction of antiviral immunity by DDX3-ligand abortive HIV-1 RNA. Here we synthesized a 58 nucleotide-long capped RNA (HIV-1 Cap-RNA_58_) that mimics abortive HIV-1 RNA transcripts. HIV-1 Cap-RNA_58_ induced potent type I IFN responses in monocyte-derived DCs, monocytes, macrophages and primary CD1c^+^ DCs. Compared with RLR agonist poly-I:C, HIV-1 Cap-RNA_58_ induced comparable levels of type I IFN responses, identifying HIV-1 Cap-RNA_58_ as a potent trigger of antiviral immunity. In monocyte-derived DCs, HIV-1 Cap-RNA_58_ activated the transcription factors IRF3 and NF-κB. Moreover, HIV-1 Cap-RNA_58_ induced DC maturation and the expression of pro-inflammatory cytokines. HIV-1 Cap-RNA_58_-stimulated DCs induced proliferation of CD4^+^ and CD8^+^ T cells and differentiated naïve T helper (T_H_) cells toward a T_H_2 phenotype. Importantly, treatment of DCs with HIV-1 Cap-RNA_58_ resulted in an efficient antiviral innate immune response that reduced ongoing HIV-1 replication in DCs. Our data strongly suggest that HIV-1 Cap-RNA_58_ induces potent innate and adaptive immune responses, making it an interesting addition in vaccine design strategies.

## Introduction

Evoking potent and tailored antiviral responses by the host is paramount in combating viral infections (1). Dendritic cells (DCs) induce antiviral immune responses by recognizing invading viruses via pattern recognition receptors (PRRs). PRR triggering by viral pathogen-associated molecular patterns (PAMPs) induces DC maturation and activation as well as differentiation of naïve T cells (2–4). Certain PRRs such as the RIG-I-like receptors (RLRs) induce strong antiviral innate immune responses initiated by expression of type I interferon (IFN) responses. RLRs are cytosolic PRRs and sense virus infection by detection of specific viral RNA structures (5, 6). RIG-I (DDX58) and MDA5 are two well-described RLRs and important in antiviral immunity to e.g., Influenza viruses, Dengue virus, and West Nile virus (7–9). RIG-I recognizes both uncapped 5′ppp single stranded (ss) RNA and short double stranded (ds) RNA, whereas MDA5 senses long dsRNA (7, 10, 11). Upon activation, RIG-I and MDA5 engage with mitochondrial antiviral protein MAVS, leading to MAVS multimerization and subsequent recruitment of adaptor molecule TRAF3. TRAF3 mediates recruitment of serine/threonine-protein TANK-binding kinase 1 (TBK1) and IκB kinase ε (IKKε), resulting in activation of transcription factors IRF3 and NF-κB, leading to type I IFN and cytokine transcription (12, 13). Autocrine and paracrine ligation of IFNβ to the heterodimeric transmembrane IFN receptor (IFNAR) on the cell surface ultimately results in transcription of a broad spectrum of interferon-stimulated genes (ISGs), of which many exhibit strong antiviral activity (14–18). NF-κB activation regulates transcriptional activation of a plethora of cytokine genes, including IL-1β and TNF that are important in innate immunity (19). In addition, IFNβ is crucial in driving IL-27 synthesis and thereby CD8^+^ T cell-dependent adaptive immune responses (20). Moreover, both IRF3 and NF-κB are involved in the induction of cytokines important in T helper (T_H_) 1 differentiation (21–23).

Recently, the cytosolic DEAD-box RNA helicase 3 (DDX3) that resembles cytosolic DEAD box helicase RIG-I and MDA5, was shown to function as a PRR for human immunodeficiency virus 1 (HIV-1) (24–27). DDX3 acts as a host factor for various viruses including hepatitis B and C virus and West Nile virus, facilitating virus replication (8, 28, 29). Thus, DDX3 is an important host factor for viruses and its function as a PRR might prevent escape of viruses from DDX3.

DDX3 is a well-known host factor required for HIV-1 propagation due to its role in transport of viral *Tat* mRNA and subsequent formation of translation initiation complexes (30–32). Upon initiation of HIV-1 infection, deficient transcription elongation leads to formation of prematurely aborted RNAs (33, 34). Interestingly, DDX3 senses these abortive HIV-1 RNA transcripts, leading to MAVS-dependent type I IFN responses, indicating that DDX3 is a viral PRR for HIV-1 (27).

Abortive HIV-1 RNAs are generated during the early steps of HIV-1 transcription, consisting of an HIV-1-specific complex secondary RNA hairpin-like structure (TAR loop) and a 5′cap, but lacking a poly A tail (33, 34). The complex structure of the TAR loop, together with the 5′cap is required for binding to DDX3, while the absence of the poly A tail prevents engagement of DDX3 with the cellular translational machinery (30). Gringhuis et al. have shown that DDX3 is an important PRR that senses abortive HIV-1 RNA transcripts upon HIV-1 infection. However, during infection of DCs, HIV-1 hijacks DC-SIGN function to block MAVS signaling, thereby preventing type I IFN and cytokine responses, and subsequently preventing the induction of antiviral innate and adaptive immune responses (27, 35). Due to this viral inhibition mechanism, the breadth and potency of abortive HIV-1 RNAs in inducing antiviral immunity remains elusive.

To characterize the role of abortive HIV-1 RNA in establishing innate and adaptive immune responses without interference due to DC-SIGN inhibition, we developed a synthetic 5′capped HIV-1 RNA of 58 nucleotides that mimics the naturally occurring abortive HIV-1 RNA (HIV-1 Cap-RNA_58_). Our data strongly suggest that HIV-1 Cap-RNA_58_ induces potent type I IFN responses in monocyte-derived DCs, macrophages as well as primary CD1c^+^ DCs. Furthermore, HIV-1 Cap-RNA_58_ is a potent stimulus that induces both innate and adaptive immune responses in monocyte-derived DCs. HIV-1 Cap-RNA_58_-dependent induction of DC maturation and cytokine secretion leads to T_H_2 differentiation. Notably, HIV-1 Cap-RNA_58_ responses inhibited ongoing HIV-1 infection. Our data further define the importance of sensing abortive HIV-1 transcripts to evoke strong antiviral immunity and provide a rationale for using HIV-1 Cap-RNA_58_ in vaccine design strategies.

## Materials and Methods

### RNA Constructs

The synthetic abortive HIV-1 RNAs were designed based on the HIV-1 genome. HIV-1 Cap-RNA_58_ consists of nucleotides 1–58 from the HIV-1 genome, including a 5′m^7^GTP cap but lacking the poly A tail. HIV-1 Cap-RNA_630_ consists of nucleotides 1–630 and also contains the 5′m^7^GTP cap and is lacking the poly A tail. The 5′m^7^GTP cap was incorporated using co-capping of 5′m^7^GTP during *in vitro* transcription (IVT) (Biosynthesis, Table S1) as previously described (27). As a control RNA, 1–58 nucleotides were synthesized lacking the 5′cap (HIV-1 control RNA_58_). HIV-1 Cap-RNA_58_ and HIV-1 control RNA_58_ structures were predicted with the MFOLD program.

### Myeloid Cell Stimulations

This study was performed according to the Amsterdam University Medical Centers, location AMC Medical Ethics Committee guidelines and all donors gave written informed consent in accordance with the Declaration of Helsinki. CD14^+^ monocyte isolation and subsequent generation of monocyte-derived DCs was performed as previously described (36). CD14^+^ monocytes were cultured in IMDM supplemented with 10% FCS, 10 U/mL penicillin and 10 mg/mL streptomycin (IMDM complete, Invitrogen) O/N at 37°C 5% CO_2_ for monocyte stimulations or cultured for 6 days in IMDM complete supplemented with GM-CSF (800 U/mL, Invitrogen) to obtain monocyte-derived macrophages. Stimulations were performed on day 1 (monocytes) or day 6 (monocyte-derived macrophages and DCs). CD1c^+^ DCs were isolated using human CD1c^+^ dendritic cell isolation kit (Miltenyi Biotec) according to manufacturer's instructions. Cells were stimulated with synthetic HIV-1 RNAs (1 nM, Biosynthesis) complexed with transfection reagent lyovec (InvivoGen), polyinosinic:polycytidylic acid complexed with lyovec (poly-I:C, 4 μg/mL, InvivoGen) and lipopolysaccharide (LPS) *Salmonella enterica* serotype typhimurium (10 ng/mL, Sigma). Poly-I:C concentration series were performed with molecular weight ranges from 223801.1 to 895204.4 g/mol. Cells were pre-incubated with blocking IFNα/βR antibodies (clone MMHAR2, 20 μg/mL) and BAY 11–7082 (2 μM) for 30 min and 2 h, respectively.

### RNA Interference

RNA interference was performed using the Neon Transfection System according to manufacturer's protocol (Thermo Fisher). On day 4 of monocyte-derived DC cultures, cells were washed with PBS, resuspended in buffer R (Thermo Fisher) and divided according to the different short interfering (si) SMARTpool RNAs (all from Dharmacon). siDDX3 (M-006874-01), siMAVS (M-024237-02) or siNon-Target as a control (D-001206-13) were added to DC-buffer R mixtures and transfection of DCs with the siRNAs was achieved by subjecting them to 1,500V for 20 ms. Transfected cells were seeded in 24-wells plates in RPMI 1640 with 10% FCS (Invitrogen) and 2 mM L-glutamine (Lonza), without antibiotics. After 48 h, viable cells were harvested, washed and seeded in a 96-wells round bottom plate and incubated overnight at 37°C, 5% CO_2_. Seventy-two hours after transfection, silencing of expression of target proteins in DCs was confirmed by quantitative real-time PCR and flow cytometry (Figure S1) and cells were stimulated as previously described.

### Quantitative Real-Time PCR

mRNA was extracted using an mRNA capture kit (Roche) and was reverse transcribed to cDNA using a reverse transcriptase kit (Promega). Quantitative real-time PCR was performed on an ABI 7500 Fast Real-Time PCR detection system (Applied Biosystems) using SYBR Green (Thermo Fisher), with primers that were designed using Primer Express 2.0 (Applied Biosystems, Table S2). Expression of genes of interest were normalized to expression of household gene *GAPDH*, according to the formula Nt = 2^Ct(GAPDH)−Ct(target)^. For each donor, expression levels induced upon stimulation with HIV-1 Cap-RNA_58_ were set as 1.

### Flow Cytometry

DCs were stimulated for 24 or 48 h, fixed with 4% paraformaldehyde (pFA) and stained with PE-conjugated anti-CD80 (1:12.5, 557227, BD pharmingen), allophycocyanin-conjugated CD83 (1:25, 551073, BD Pharmingen), FITC-conjugated anti-CD86 (1:25, 555657, BD Pharmingen), PE-conjugated anti-HLA-DR (1:25, 555812, BD Pharmingen), or PE-Cy7-conjugated anti-CD40 (1:100, 2165055, Sony Biotechnology). Expression levels after RNA interference were determined using anti-DDX3 or anti-MAVS (1:50, 2635S or 3993S, Cell Signaling) followed by PE-conjugated donkey anti-rabbit (1:200, Jackson Immuno Research). HIV-1 infection levels were assessed using anti-p24 (1:200, KC57-RD1, Beckman Coulter). Flow cytometric analysis was performed using the FACSCanto II (BD Biosciences) and FlowJo software v10.

### p65 and IRF3 Translocation

DCs were stimulated for 4 h, fixed with 4% pFA and permeabilized with 0.2% Triton X-100 in PBS. p65 was stained with anti-p65 (1:50, 8242S, Cell Signaling) and IRF3 with anti-IRF3 (1:50, 4302S, Cell Signaling), followed by a secondary donkey anti-rabbit labeled with Alexa-546 (1:400, Invitrogen) and 1 μg/mL Hoechst (Invitrogen) and cellular localization was visualized with a 100x magnification, using Leica DM6 B upright microscope. Analysis was performed with LAS X Navigator software. Nuclear extracts (NE) were prepared 4 h after DC stimulation, using the NucBuster protein extraction kit (Novagen). Twenty micrograms of NE per sample was used to detect nuclear p65 or IRF3 using the TransAM NF-κB-p65 and IRF3 kits (Active Motif). OD450 nm values were measured using BioTek Synergy HT.

### Elisa

DC supernatants were harvest 24 or 48 h after stimulation and secretion of TNF, IL-6, and IL-12p70 protein (eBiosciences) was measured by ELISA as described by manufacturer. OD450 nm values were measured using BioTek Synergy HT.

### T Cell Proliferation and Differentiation

To assess T cell proliferation levels, DCs were primed with stimuli and cocultured in a 1:4 ratio with allogeneic peripheral blood lymphocytes (PBLs) isolated from buffy coats of healthy donors (Sanquin) labeled with CellTrace Violet (Thermo Fisher). On day 3, IL-2 was added (10 U/mL, Chiron). After 5 days, cells were fixed with 4% pFA and stained with FITC-conjugated anti-CD3 (1:100, 11003642, ebioscience), PerCP-Cy5.5-conjugated anti-CD4 (1:20, 332772, BD Biosciences), PE-Cy7-conjugated anti-CD8 (1:100, 25008742, eBioscience) and fixable viability dye eFluor780 (1:4000, 65-0865-14, ebioscience). Proliferation was assessed in viable CD4^+^ and CD8^+^, CD3^+^ T cells. To examine T_H_ cell differentiation, DCs were primed for 48 h with stimuli or LPS (10 ng/mL) in combination with IFNγ (1,000 U/mL, u-CyTech) or PGE_2_ (1 μM, Sigma), as positive controls for T_H_ 1 and 2 skewing, respectively. DCs were cocultured in the presence of *Staphylococcus aureus* enterotoxin B (10 pg/mL, Sigma) in a 1:4 ratio with allogeneic naïve T cells that were isolated from PBMCs or PBLs from buffy coats with a human CD4^+^ T cell isolation kit II (Miltenyi Biotec), with PE-conjugated anti-CD45RO (Dako) and anti-PE beads (Miltenyi Biotec) according to manufacturer's instructions. On day 5, IL-2 was added (10 U/mL, Chiron). After 11–13 days, resting T cells were restimulated with PMA (10 ng/mL, Sigma) and ionomycin (1 mg/mL, Sigma) for 6 h and treated with brefeldin A (10 μg/mL, Sigma) for the final 4 h. Cells were fixed for flow cytometry analysis with 4% pFA, permeabilized using 0.1% saponin in PBS and stained with FITC-conjugated anti-IFNγ (1:5, 340449, BD Biosciences) and allophycocyanin-conjugated anti-IL-4 (1:25, 554486, BD Biosciences) to determine T_H_ 1 and 2 differentiation, respectively. Cells were analyzed using the FACS Canto II (BD Biosciences) and FlowJo software v10.

### Virus and Infection

DCs were infected with R5 HIV-1 strain NL4.3 BaL. NL4.3 BaL was produced as described previously (34, 37). For DC infection, a multiplicity of infection (MOI) of 0.1–0.2 was used, depending on the virus batch. DCs were infected for 24 h, washed extensively and left in the presence of HIV-1 control RNA or HIV-1 Cap-RNA_58_ for 5 days, after which intracellular p24 levels were measured using flow cytometry to determine infection.

### Statistical Analysis

Statistics were performed using Student's *t*-test for paired (BAY 11-7082 inhibitor, IL-6 and TNF ELISAs, proliferation assay) and unpaired observations (all other experiments) using GraphPad version 8. Statistical significance was set at *P* < 0.05.

## Results

### HIV-1 Cap-RNA_58_ Induces Type I IFN Responses in Various Myeloid Cells

**Figure 1:**
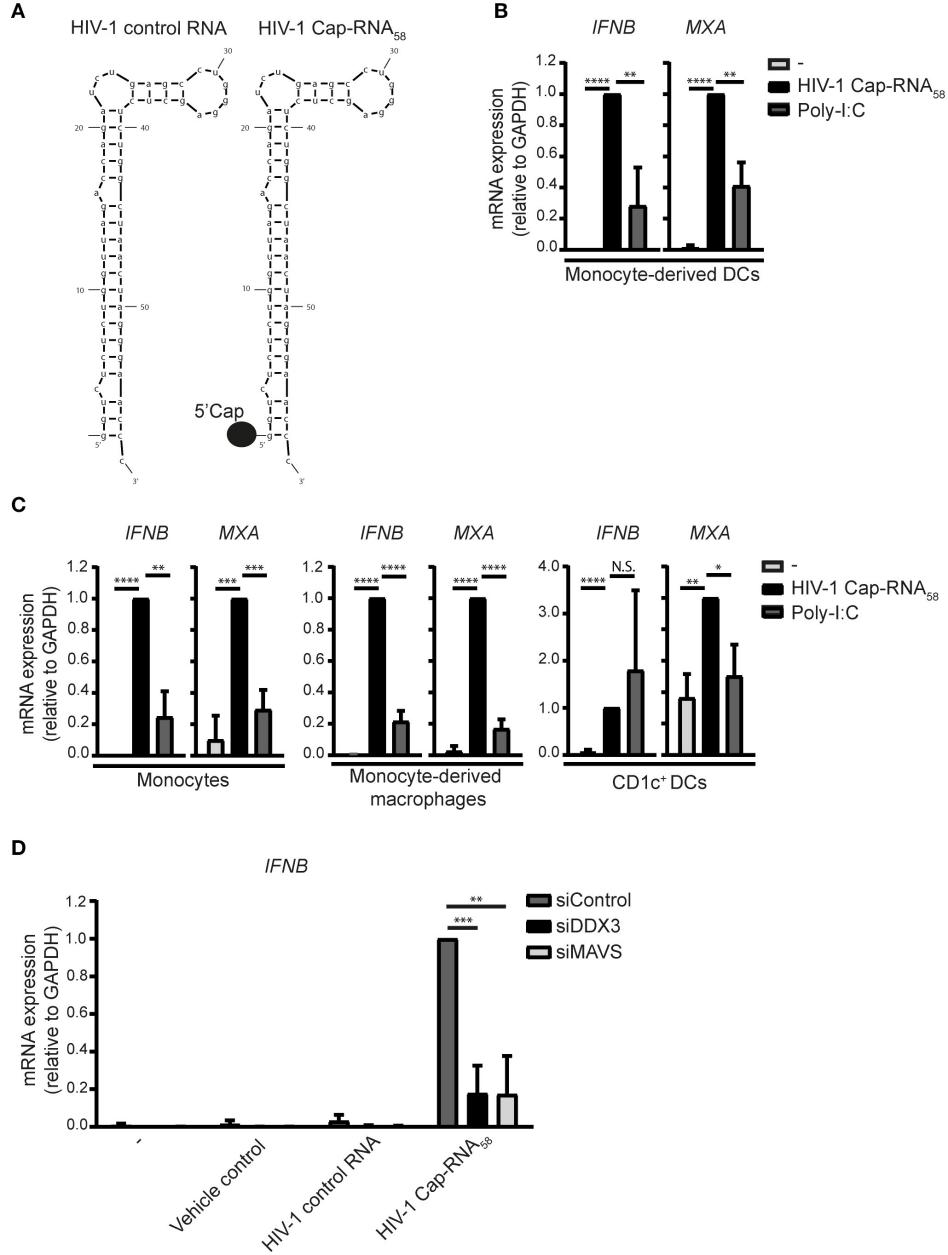
HIV-1 Cap-RNA_58_ induces type I IFN responses in various myeloid immune cells. **(A)** Schematic sequence of HIV-1 control RNA and HIV-1 Cap-RNA_58_. **(B)** Monocyte-derived DCs, **(C)** CD14^+^ monocytes, monocyte-derived macrophages and CD1c^+^ DCs were stimulated with 1 nM HIV-1 Cap-RNA_58_ or poly-I:C complexed with lyovec (vehicle control), and both *IFNB* and *MXA* expression was measured by quantitative real-time PCR after 10 h (monocyte-derived DCs, CD14^+^ monocytes and monocyte-derived macrophages) or 8 h (CD1c^+^ DCs). Values were calculated relative to *GAPDH*. HIV-1 Cap-RNA_58_-induced responses were set as 1. **(D)** DCs were treated with vehicle control, 1 nM HIV-1 control RNA, and 1 nM HIV-1 Cap-RNA_58_, 72 h after silencing of DDX3 and MAVS. *IFNB* mRNA expression was measured after 2 h of stimulation by quantitative real-time PCR and mRNA expression was relative to *GAPDH*. HIV-1 Cap-RNA_58_-induced *IFNB* responses in siControl-treated DCs was set as 1. Data are representative of collated data of three **(B–D)** or four **(C**, monocyte-derived macrophages) donors (mean ± s.d.). *P < 0.05, **P < 0.01, ***P < 0.001, ****P < 0.0001, Student's *t*-test. NS, not significant.

We used a synthetic RNA that mimics abortive HIV-1 RNAs: this synthetic HIV-1 Cap-RNA_58_ consists of the first 58 nucleotides common to all HIV-1 transcripts and contains a 5′cap while lacking a poly A tail (Table S1, Figure 1A). We investigated whether HIV-1 Cap-RNA_58_ induced type I IFN responses in monocyte-derived DCs (DCs) by treating DCs with HIV-1 Cap-RNA_58_ complexed with transfection reagent lyovec (vehicle control) to facilitate cytoplasmic delivery (27). HIV-1 Cap-RNA_58_ induced strong *IFNB* transcription in DCs after 10 h of stimulation (Figure 1B). Expression levels of interferon-stimulated gene (ISG) Myxovirus resistance protein 1 (MxA) transcripts were significantly induced by HIV-1 Cap-RNA_58_ in DCs as well when compared to untreated DCs (Figure 1B). HIV-1 Cap-RNA_58_-induced type I IFN levels were compared to those observed with RLR agonist poly-I:C. After equalizing the amount of molecules of both HIV-1 Cap-RNA_58_ and poly-I:C (Figure S1), HIV-1 Cap-RNA_58_-induced type I IFN were significantly higher than poly-I:C-induced levels after 10 h of stimulation (Figure 1B). We next investigated whether HIV-1 Cap-RNA_58_ activated responses in primary myeloid cells. Notably, treatment of CD14^+^ monocytes and monocyte-derived macrophages with HIV-1 Cap-RNA_58_ induced type I IFN responses 10 h after stimulation, whereas primary blood CD1c^+^ DCs showed type I IFN responses 8 h after stimulation. HIV-1 Cap-RNA_58_-induced type I IFN levels were higher than or comparable to poly-I:C-induced type I IFN responses (Figure 1C). To assess the specificity of HIV-1 Cap-RNA_58_ for DDX3 and MAVS-dependent signaling, both DDX3 and MAVS were silenced in DCs by RNA interference (RNAi) (Figure S2). Silencing of either DDX3 or MAVS expression completely abrogated *IFNB* induced by HIV-1 Cap-RNA_58_, 2 h after stimulation (Figure 1D). Both vehicle control and HIV-1 control RNA did not induce *IFNB* expression (Figures 1A,D). These data indicate that HIV-1 Cap-RNA_58_ triggers type I IFN in a variety of myeloid cells via DDX3 and MAVS, and that the 5′cap is required for sensing by DDX3.

### HIV-1 Cap-RNA_58_ Induces DC Maturation

**Figure 2:**
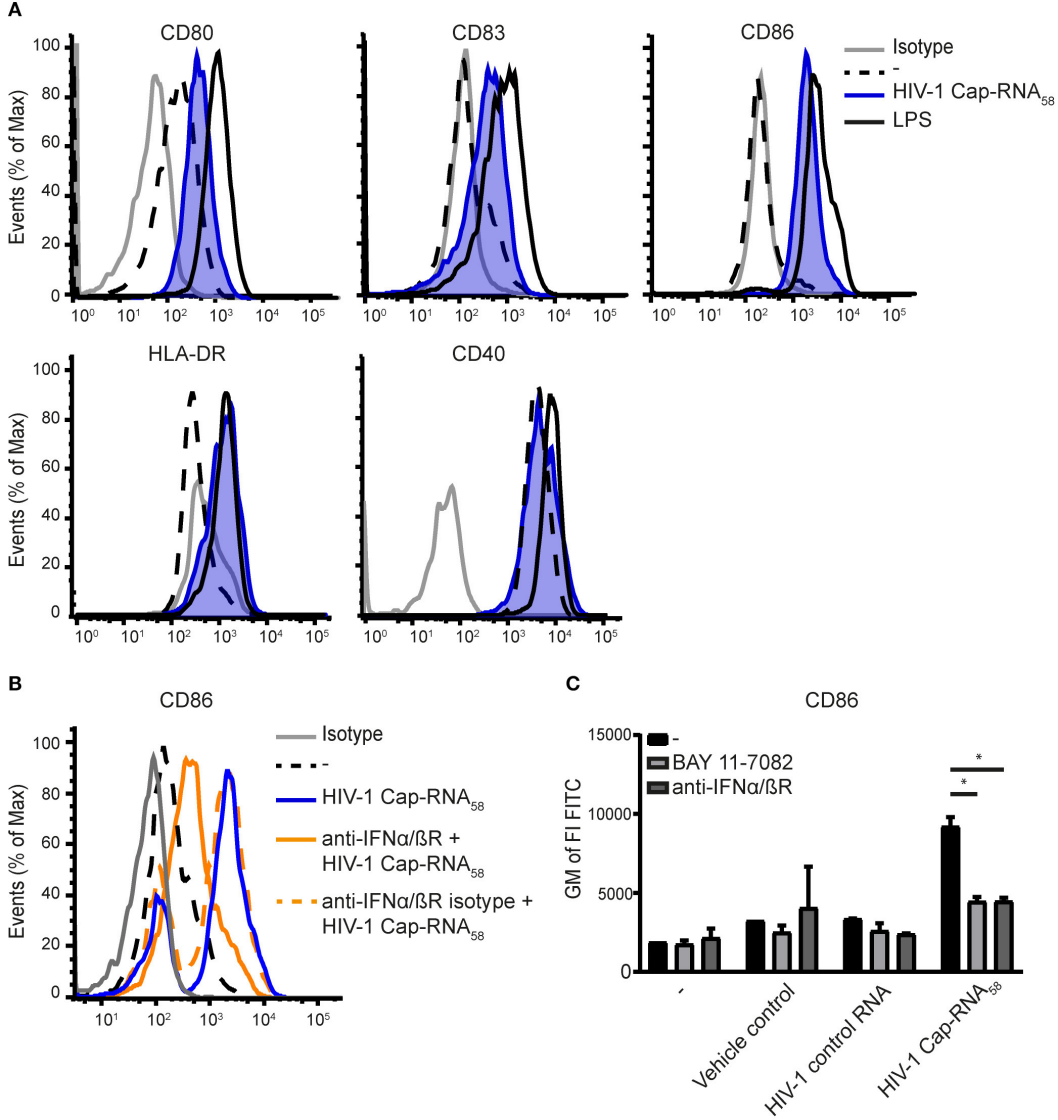
HIV-1 Cap-RNA_58_ induces DC maturation. **(A)** DCs were not treated or treated with 1 nM HIV-1 Cap-RNA_58_. DCs were fixed and expression levels of CD80, CD83, CD86, and CD40 (24 h after stimulation) and HLA-DR (48 h after stimulation) were measured by flow cytometry. **(B)** DCs were stimulated with 1 nM HIV-1 Cap-RNA_58_ in the presence or absence of blocking IFNα/βR antibodies or **(C)** with blocking IFNα/βR antibodies and BAY 11-7082 inhibitor. Geometric mean of the fluorescent intensity (GM of FI) of FITC was shown to indicate CD86 expression levels. Data are representative of at least three donors **(A–C)** from different experiments (mean ± s.d.). *P < 0.05, Student's *t*-test.

To determine whether HIV-1 Cap-RNA_58_ induces adaptive immunity, we first examined the expression levels of costimulatory molecules CD80, CD83, and CD86 after stimulation with HIV-1 Cap-RNA_58_, by flow cytometry. HIV-1 Cap-RNA_58_ induced expression of CD80, CD83, and CD86 compared to unstimulated DCs, albeit to a lesser extent than LPS (Figure 2A). HIV-1 Cap-RNA_58_ induced expression levels of activation marker HLA-DR to a similar extent as LPS, whereas the expression levels of costimulatory molecule CD40 remained unaffected in contrast to LPS stimulation (Figure 2A). To investigate the role of IFNβ signaling in DC maturation, we neutralized IFNβ signaling by treatment with blocking IFNα/βR antibodies. CD86 induction by HIV-1 Cap-RNA_58_ was partially but significantly blocked by blocking IFNα/βR antibodies (Figures 2B,C), suggesting that type I IFN induction increases CD86. Besides type I IFN-dependent CD86 expression, expression of costimulatory molecules can also be induced by the transcription factor NF-κB (38, 39). To test whether CD86 expression is also NF-κB dependent, we blocked NF-κB activation using BAY 11-7082, a small molecule inhibitor for IκBα, as release of the inhibitory protein IκBα from the NF-κB dimer within the cytoplasm is mandatory for NF-κB activation (40). BAY 11-7082 treatment significantly reduced HIV-1 Cap-RNA_58_-induced CD86 expression (Figure 2C). Our results imply that HIV-1 Cap-RNA_58_ treatment leads to IFNβ- and NF-κB-dependent DC maturation.

### HIV-1 Cap-RNA_58_ Activates IRF3 and NF-κB p65

**Figure 3:**
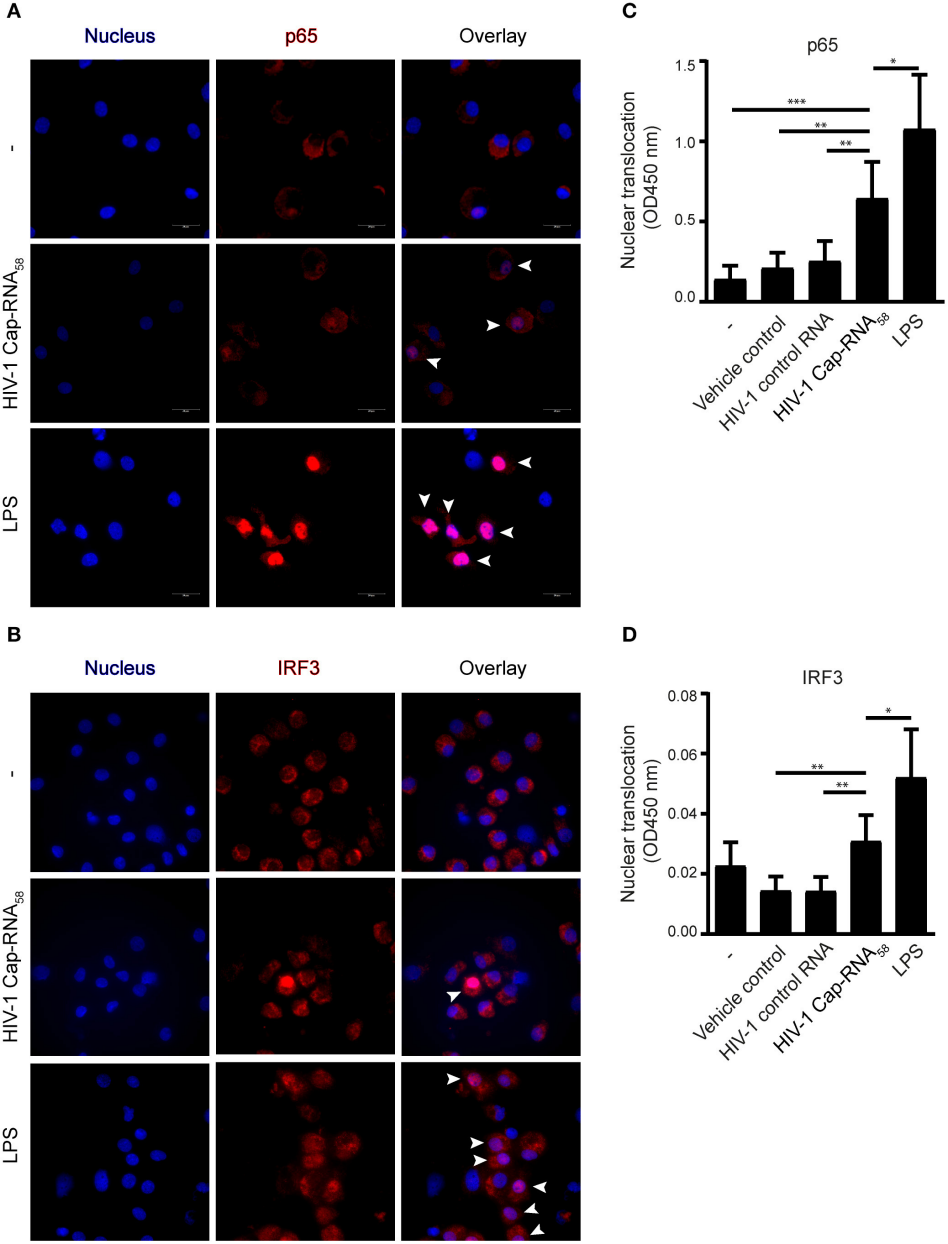
HIV-1 Cap-RNA_58_ activates IRF3 and NF-κB p65. **(A,B)** DCs were not treated or treated with 1 nM HIV-1 Cap-RNA_58_ or LPS. After 4 h, cells were fixed, permeabilized and stained with Hoechst (to visualize the nucleus) and with anti-p65 or anti-IRF3 and secondary rabbit-Alexa-546-labeled antibodies and visualized using fluorescent microscopy, 100x magnification. **(C)** DCs were not treated or treated with vehicle control (lyovec), 1 nM HIV-1 control RNA, 1 nM HIV-1 Cap-RNA_58_ or LPS and nuclear extracts were obtained 4 h after stimulation. Twenty micrograms of nuclear extract was analyzed with a transAM assay to determine nuclear translocation of p65 **(D)** or IRF3, using OD450 nm. Data are representative of three donors of different experiments **(A)** or are collated data of six **(C)** or five **(D)** donors (mean ± s.d.). *P < 0.05, **P < 0.01, ***P < 0.001, Student's *t*-test.

We next investigated whether HIV-1 Cap-RNA_58_ activated transcription factors IRF3 and NF-κB, known to be involved in transcriptional regulation of a plethora of cytokine and other genes required for the orchestration of innate and adaptive immune responses by DCs (14, 15, 19). DCs were treated with HIV-1 Cap-RNA_58_ or LPS and translocation of p65, one of the primary active subunits within the dimeric NF-κB transcription factor family, was analyzed by immunofluorescence microscopy. Similarly, as was observed for IRF3, HIV-1 Cap-RNA_58_ induced p65 translocation to the nucleus, albeit to a lesser extent than observed for LPS-treated DCs (Figures 3A,B). We next quantified nuclear translocation of p65 and IRF3 using a transcription factor binding assay using nuclear extracts from HIV-1 Cap-RNA_58_-activated DCs. HIV-1 Cap-RNA_58_ induced significant translocation of the p65 unit, 4 h after stimulation, in contrast to HIV-1 control RNA (Figure 3C). Similarly, we quantified the nuclear translocation of IRF3, which was also detected in HIV-1 Cap-RNA_58_-activated but not HIV-1 control RNA-treated DCs (Figure 3D). Thus, these data strongly indicate that HIV-1 Cap-RNA_58_ activates both IRF3 and NF-κB, implying that it can play a significant role in establishing innate and adaptive immune responses.

### HIV-1 Cap-RNA_58_ Induces Expression of Pro-Inflammatory Cytokines

**Figure 4:**
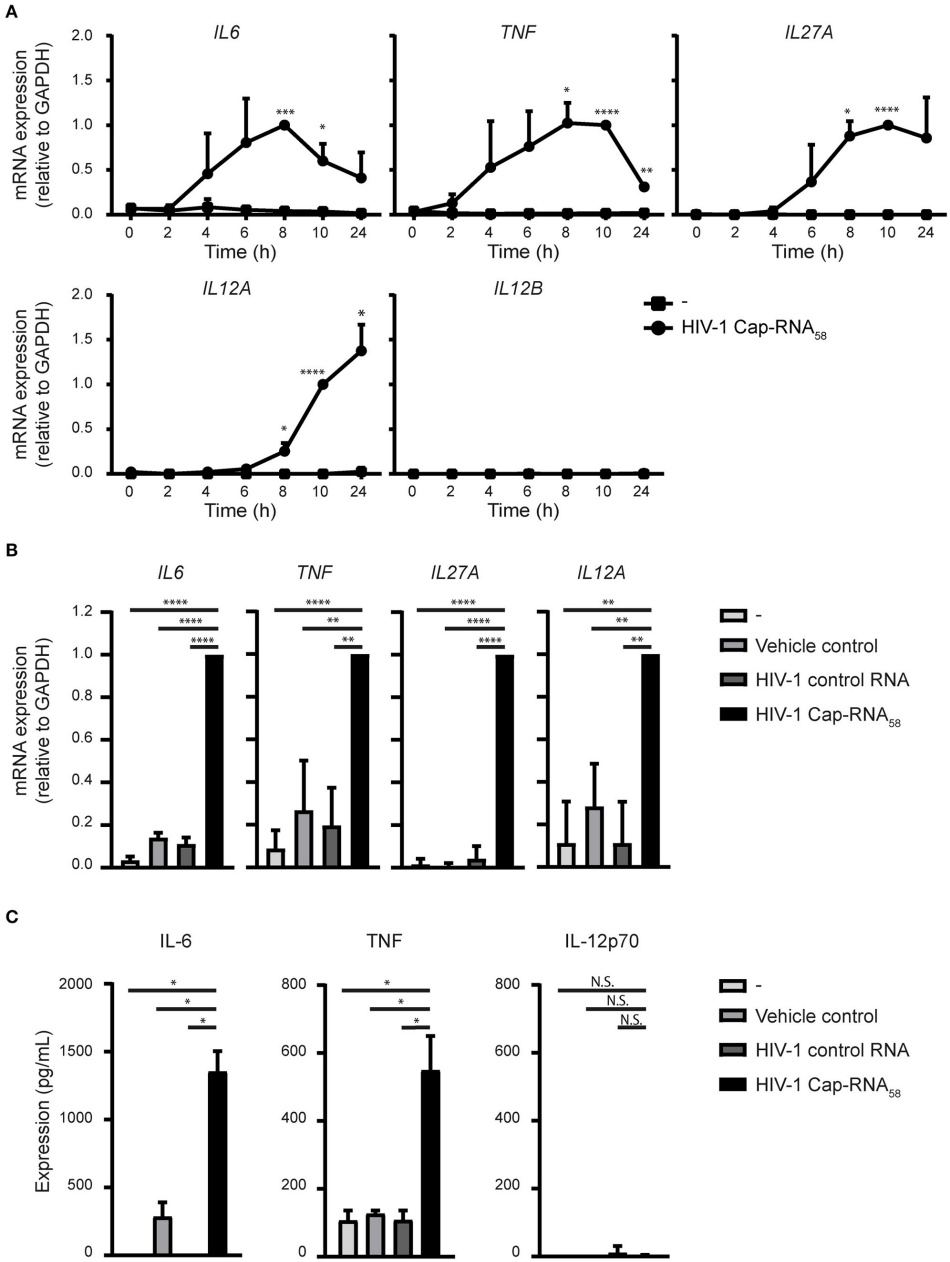
HIV-1 Cap-RNA_58_ induces expression of pro-inflammatory cytokines. **(A)** DCs were not treated or stimulated with 1 nM HIV-1 Cap-RNA_58_ and mRNA expression levels of *IL6, TNF, IL27A, IL12A*, and *IL12B* were measured every 2 h with quantitative real-time PCR, relative to *GAPDH*. HIV-1 Cap-RNA_58_-induced responses at 8 h were set as 1 for *IL6* and at 10 h for all other cytokines genes. **(B)** DCs were left untreated, treated with vehicle control or 1 nM HIV-1 control RNA or stimulated with 1 nM HIV-1 Cap-RNA_58_ and mRNA expression levels of *IL6, TNF, IL27A*, and *IL12A* were measured at peak level with quantitative real-time PCR, relative to *GAPDH*. HIV-1 Cap-RNA_58_-induced responses at 8 h were set as 1 for *IL6* and *TNF*, at 10 h for *IL27A* and at 24 h for *IL12A* gene expression. **(C)** DCs were left untreated, treated with vehicle control or 1 nM HIV-1 control RNA or stimulated with 1 nM HIV-1 Cap-RNA_58_ and supernatant was harvested after 24 or 48 h, to determine expression levels of IL-6 (48 h), TNF (24 h), and IL-12p70 (48 h) with ELISA. Data are representative of collated data of three donors **(A,B)** or representative of three donors of different experiments **(C)** (mean ± s.d.). *P < 0.05, **P < 0.01, ***P < 0.001, ****P < 0.0001, Student's *t*-test. NS, not significant.

We next assessed whether treatment of HIV-1 Cap-RNA_58_ triggered cytokine expression in DCs. HIV-1 Cap-RNA_58_ significantly induced expression of the pro-inflammatory cytokines IL-6 and TNF at both mRNA and protein level, compared to vehicle control and HIV-1 control RNA-treated DCs (Figures 4A–C). Interestingly, HIV-1 Cap-RNA_58_ treatment of DCs did not induce mRNA expression of *IL1B, IL8, IL10*, and *IL23A* (data not shown). mRNA expression levels of *IL6* and *TNF* peaked at 10 h, while the ISG *IL27A*, which encodes IL-27p28, a subunit of IL-27, peaked at 8 h (Figure 4A). At peak level, the expression levels of HIV-1 Cap-RNA_58_-induced *IL6, TNF, IL27A*, and *IL12A* mRNA were significantly increased compared to both vehicle control and HIV-1 control RNA (Figure 4B). Whereas, *IL12A* transcription was induced upon treatment with HIV-1 Cap-RNA_58_, *IL12B* mRNA could not be detected (Figure 4A). In line with the lack of expression of the *IL12B* subunit, no bioactive IL-12p70 protein could be detected in the supernatant of DCs after HIV-1 Cap-RNA_58_ treatment (Figure 4C). Thus, HIV-1 Cap-RNA_58_ induces a specific cytokine program primarily directed at pro-inflammatory conditions.

### Different Viral HIV-1 RNAs Induce Similar Levels of Type I IFN and Pro-Inflammatory Cytokines

**Figure 5:**
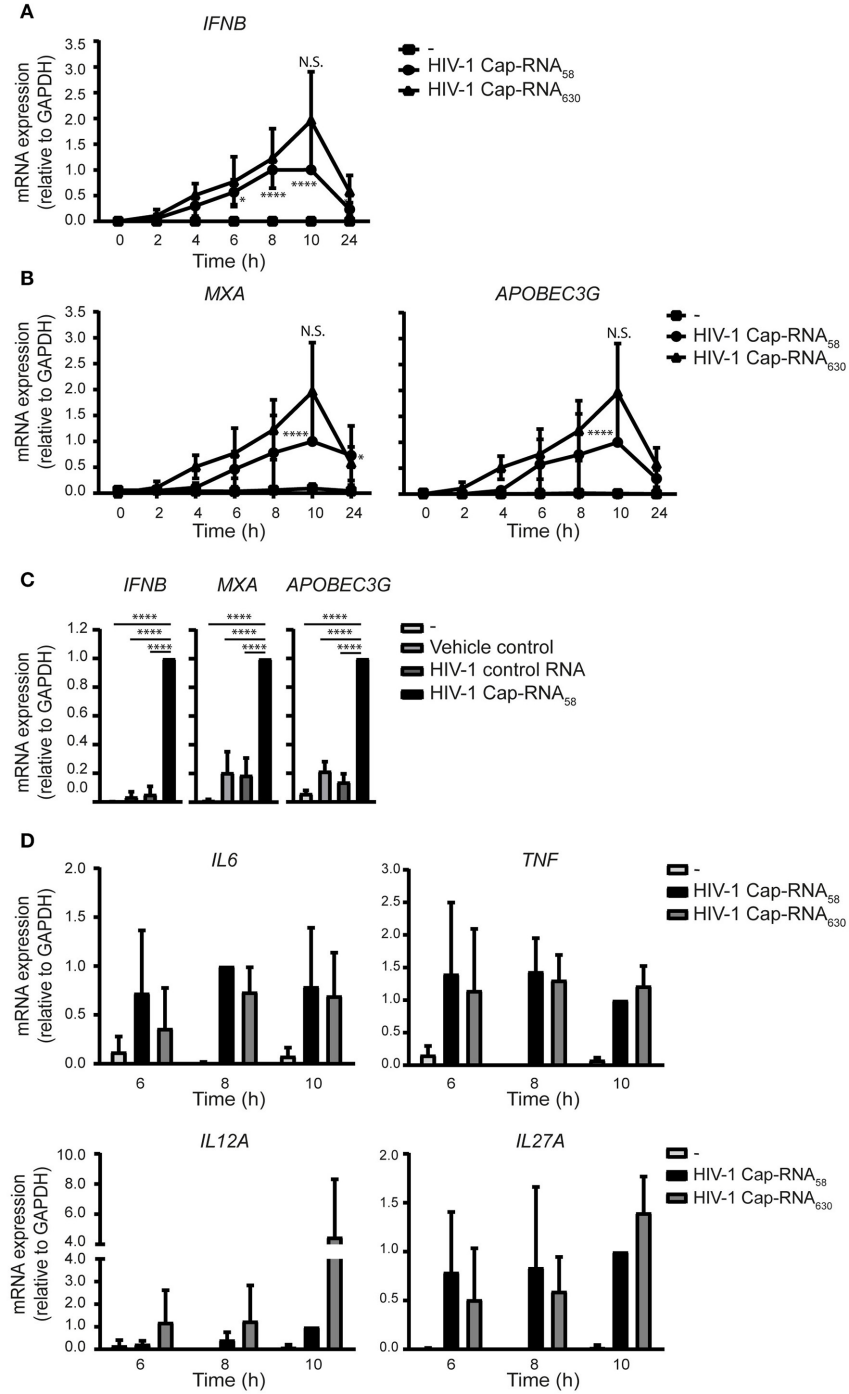
Different viral HIV-1 RNAs induce similar levels of type I IFN and pro-inflammatory cytokines. **(A)** DCs were not treated or treated with 1 nM HIV-1 Cap-RNA_58_ or 1 nM HIV-1 Cap-RNA_630_, mRNA was extracted every 2 h and analyzed for *IFNB* by quantitative real-time PCR and mRNA expression was analyzed relative to *GAPDH*. HIV-1 Cap-RNA_58_-induced responses at 10 h after stimulation were set as 1. Data point at 8 h after stimulation was also shown in Figure 1A. **(B)** *MXA* or *APOBEC3G* mRNA expression was assessed similarly. Data point at 8 h after stimulation was also shown in Figure 1B. **(C)** DCs were left untreated, treated with either vehicle control, 1 nM HIV-1 control RNA or 1 nM HIV-1 Cap-RNA_58_ and mRNA expression levels of *IFNB, MXA*, and *A3G* were measured at peak level with quantitative real-time PCR, relative to *GAPDH*. HIV-1 Cap-RNA_58_-induced responses at 10 h were set as 1. **(D)** *IL6, TNF, IL12A*, and *IL27A* mRNA expression was assessed similarly. Data are representative of collated data of three **(A,B,D)** or four **(C)** donors (mean ± s.d.). Statistical significance was determined between both HIV-1 RNA constructs and between unstimulated DCs and DCs treated with HIV-1 Cap-RNA_58_ or HIV-1 Cap-RNA_630_ **(D)**_._ *P < 0.05, ****P < 0.0001, Student's *t*-test. NS, not significant.

To assess whether the ability of HIV-1 Cap-RNA_58_ to induce type I IFN and cytokine responses is due to the short RNA construct length, we examined the ability of a longer viral HIV-1 RNA in inducing immune activation. DCs treated with a 5′capped 630 nucleotides long RNA corresponding to the *Tat* transcript, which includes the same 1–58 sequence as the HIV-1 Cap-RNA_58_ at its start, but lacking a poly A tail (HIV-1 Cap-RNA_630_) induced antiviral responses comparable to those induced by HIV-1 Cap-RNA_58_. Similar to HIV-1 Cap-RNA_58_, HIV-1 Cap-RNA_630_ induced *IFNB* 2 h after stimulation reaching a peak level after 10 h (Figure 5A). Similarly, HIV-1 Cap-RNA_630_ induced *MXA* and also another ISG *A3G* (encoding for APOBEC3G protein) transcription at similar levels as HIV-1 Cap-RNA_58_, peaking again at 10 h after stimulation (Figure 5B). At peak levels, HIV-1 Cap-RNA_58_ significantly induced *IFNB, MXA*, and *A3G* gene expression compared to DCs treated with vehicle control or HIV-1 control RNA (Figure 5C). We also assessed HIV-1 Cap-RNA_58_- and HIV-1 Cap-RNA_630_-induced *IL6, TNF, IL12A*, and *IL27A* transcription at time points that we had previously shown to have significantly enhanced expression after HIV-1 Cap-RNA_58_ treatment compared to untreated DCs and DCs treated with vehicle control and HIV-1 control RNA (Figures 4A,B). At 6, 8 and 10 h after stimulation with the two HIV-1 Cap-RNAs, we observed that both constructs induced similar levels of *IL6, TNF, IL12A*, and *IL27A* mRNA (Figure 5D). Although not significant, stimulation with HIV-1 Cap-RNA_630_ showed a trend toward increased *IL12A* expression compared to HIV-1 Cap-RNA_58_ after 6, 8, and 10 h of stimulation, and to a trend of increased *IL27A* expression after 10 h (Figure 5D). These data suggest that the length of viral RNA constructs does not affect the strength of the type I IFN and pro-inflammatory cytokine responses as long as it contains a 5′cap and the first 58 nucleotides of the HIV-1 genome that form the TAR loop. Thus, synthetic HIV-1 RNAs induce antiviral innate immune responses independent of length.

### HIV-1 Cap-RNA_58_-Activated DCs Induce T Cell Proliferation and Differentiation

**Figure 6:**
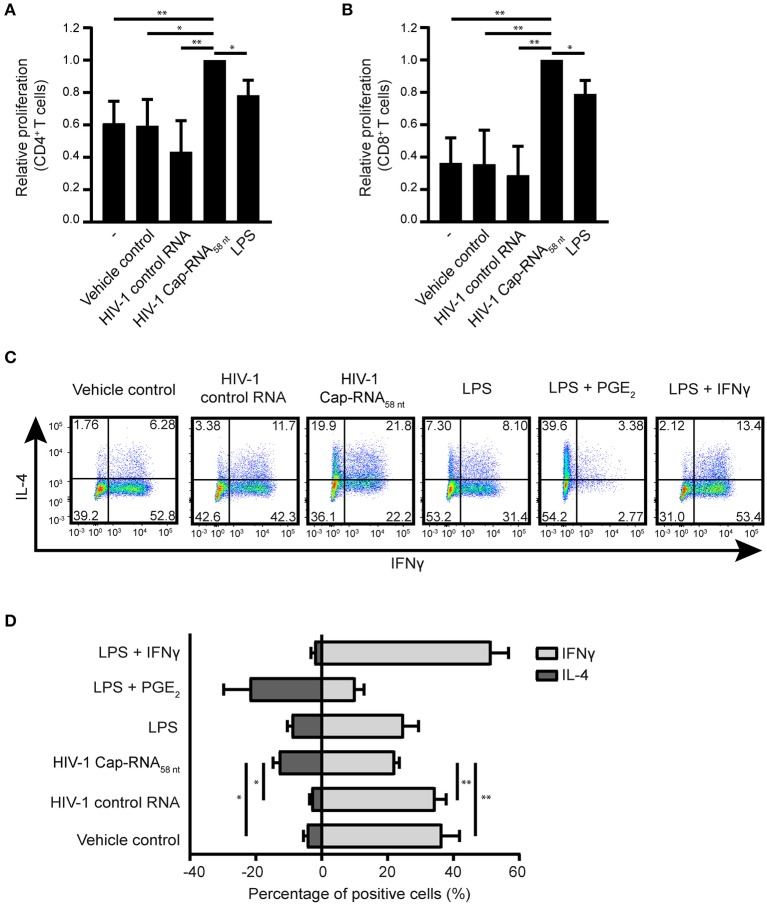
HIV-1 Cap-RNA_58_-treated DCs mediate T cell proliferation and differentiation. DCs were unstimulated or treated with 1 nM HIV-1 Cap-RNA_58_ or LPS for 48 h, cocultured with CellTrace Violet-labeled peripheral blood lymphocytes and harvested after 5 days. Proliferation was determined in **(A)** live CD3^+^ CD4^+^ cells and **(B)** CD3^+^ CD8^+^ T cells by flow cytometry. **(C,D)** DCs were treated with vehicle control, 1 nM HIV-1 control RNA, 1 nM HIV-1 Cap-RNA_58_, LPS, LPS + PGE_2_, or LPS + IFNγ for 48 h and cocultured with naïve T cells. After 11–13 days, cells were restimulated and intracellular expression levels of IFNγ (T_H_1) and IL-4 (T_H_2) were analyzed using flow cytometry. Number in plots indicate percentage of cells in the quadrant. Data are representative of collated data of three **(A,B)** or four **(D)** donors, or four donors **(C)** of different experiments (mean ± s.d.). *P < 0.05, **P < 0.01, Student's *t*-test. NS, not significant.

We next investigated the ability of HIV-1 Cap-RNA_58_-treated DCs to activate T cells. First we analyzed proliferation of CellTrace Violet-labeled peripheral blood cells (PBLs) induced by coculture for 5 days with DCs that were treated with vehicle control, HIV-1 control RNA, HIV-1 Cap-RNA_58_ or LPS. Flow cytometry analysis showed that HIV-1 Cap-RNA_58_-activated DCs enhanced proliferation of both CD4^+^ and CD8^+^ T cells compared to untreated DCs and DCs treated with vehicle control or HIV-1 control RNA (Figures 6A,B and Figure S3). We next analyzed T_H_1 and T_H_2 differentiation after DC-naïve CD4^+^ T cell cocultures by intracellular IFNγ and IL-4 expression, respectively. HIV-1 Cap-RNA_58_-activated DCs showed significant skewing toward T_H_2 differentiation compared to the DCs treated with vehicle control or HIV-1 control RNA (Figures 6C,D). As expected, positive controls LPS and PGE_2_ or LPS and IFNγ induced T_H_2 and T_H_1 responses, respectively, whereas LPS gave a mixed T_H_1/T_H_2 response (Figures 6C,D). Thus, our data show that HIV-1 Cap-RNA_58_-treated DCs induce CD4^+^ and CD8^+^ T cell activation and skew adaptive immune response toward a T_H_2 phenotype.

### HIV-1 Cap-RNA_58_ Inhibits HIV-1 Infection of DCs

**Figure 7:**
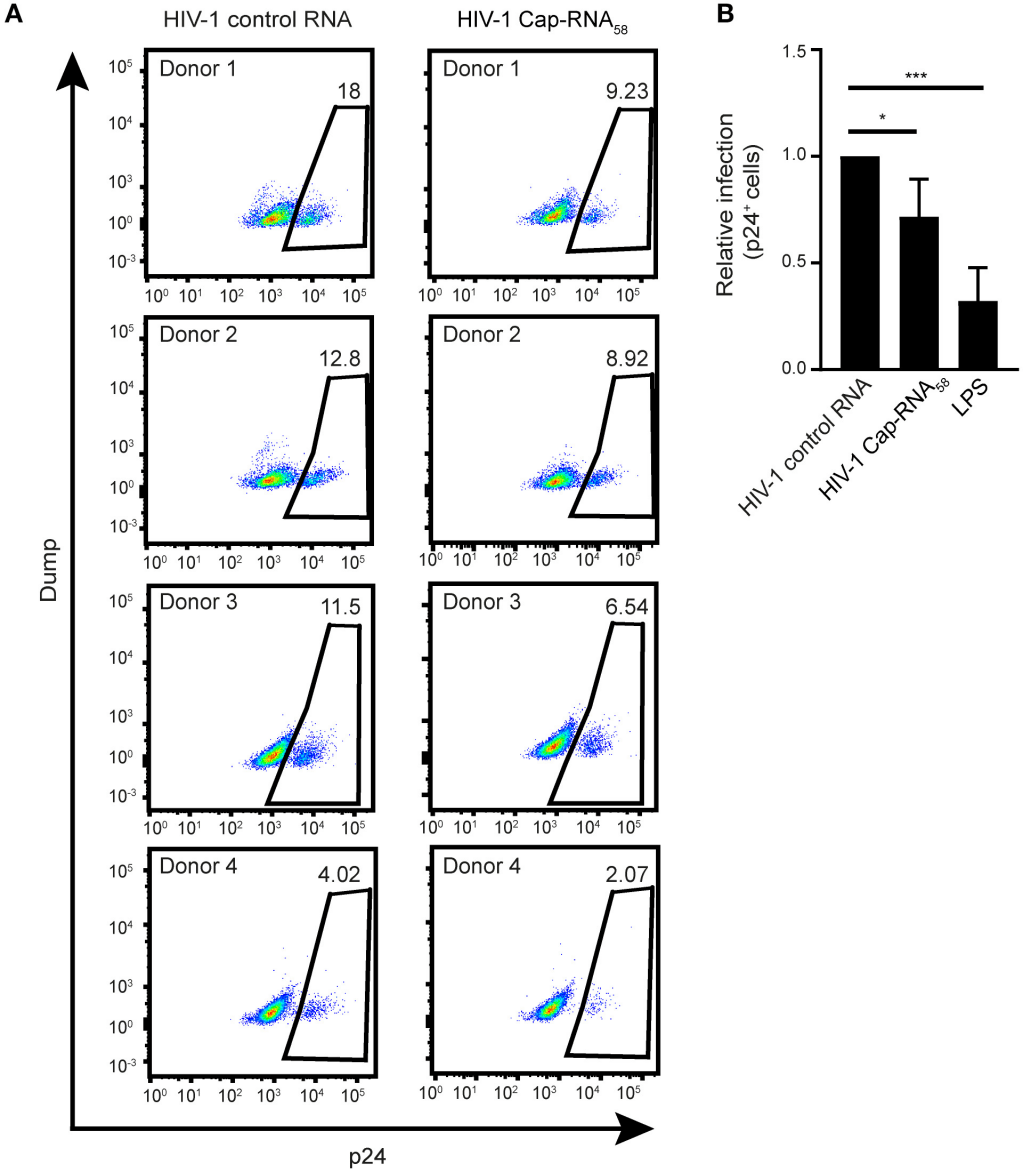
HIV-1 Cap-RNA_58_ reduces ongoing HIV-1 infection in DCs. **(A,B)** DCs were infected with NL4.3 BaL for 24 h, washed and incubated with 1 nM HIV-1 control RNA, 1 nM HIV-1 Cap-RNA_58_ or LPS. After 5 days, intracellular p24 levels were measured by flow cytometry. Data are representative of four donors **(A,B)**. (mean ± s.d.). *P < 0.05, ***P < 0.001, Student's *t*-test. NS, not significant.

To investigate whether treatment with HIV-1 Cap-RNA_58_ would block HIV-1 replication in DCs after infection, DCs were infected by R5-tropic HIV-1 NL4.3 BaL. After 24 h of ongoing HIV-1 infection the DCs were treated with HIV-1 Cap-RNA_58_ or LPS After 5 days, infection levels were assessed by measuring intracellular p24^+^ cells by flow cytometry. Although infection levels differed per donor, HIV-1 Cap-RNA_58_ decreased the percentage of HIV-1 p24^+^ DCs compared to HIV-1 control RNA-treated DCs in four different donors, albeit to a lesser extent than LPS (Figures 7A,B). These results imply that HIV-1 Cap-RNA_58_ induces a functional antiviral response that limits HIV-1 infection.

## Discussion

RNA helicase DDX3 is important for the transport of HIV-1 *Tat* mRNA as well as the formation of translation initiation complexes required for HIV-1 translation (30–32). Besides its role as a host factor for HIV-1, DDX3 also functions as a viral sensor (27). Here we investigated the potency and breadth of DDX3 ligand HIV-1 Cap-RNA_58_, a synthetic mimic of the naturally occurring abortive HIV-1 RNAs. We observed that HIV-1 Cap-RNA_58_ resulted in type I IFN responses in various myeloid cells in a DDX3- and MAVS-dependent manner. Our data further showed that HIV-1 Cap-RNA_58_ inhibited ongoing HIV-1 replication in DCs, most likely via the induced innate antiviral type I IFN responses. Moreover, HIV-1 Cap-RNA_58_ induced DC maturation and cytokine responses that led to adaptive T cell activation as well as differentiation. Thus, our data suggest that DDX3 is a PRR and shows that synthetic abortive HIV-1 RNA_58_ is immunostimulatory.

The potency of abortive HIV-1 RNA to exert an antiviral role in response to viral sensing by its natural ligand DDX3 is not well-understood. Therefore, we aimed to assess the role of abortive HIV-1 RNA in induction of innate and adaptive immune responses by using a synthetic mimic of abortive HIV-1 RNA (HIV-1 Cap-RNA_58_). Our data strongly suggest that synthetic HIV-1 Cap-RNA_58_ encapsulated in lyovec triggered DDX3 and MAVS-mediated IFNβ responses. It has been described that DDX3 has been found in complexes with RIG-I and MDA5 and might therefore induce IFNβ responses via a RIG-I or MDA5-MAVS-dependent way (41). We have previously shown in 293T cells treated with CRISPR-cas9 that depletion of RIG-I and MDA5 did not affect HIV-1 Cap-RNA_58_-induced type I IFN responses, indicating that the HIV-1 Cap-RNA_58_-induced type I IFN responses described here are generated in a DDX3-MAVS-TBK1-IRF3-dependent manner (27). Previous studies have shown that knockdown of DDX3 or the generation of phosphorylation-deficient DDX3 mutants in cell lines resulted in TBK1/IKKε-dependent decrease of the *IFNB* promotor activity, providing evidence that DDX3 is involved in the induction of *IFNB* transcription (24, 25). In line with the previous reported data obtained in cell lines, we have shown that DDX3 induces type I IFN responses in monocyte-derived DCs as well as primary monocytes, monocyte-derived macrophages and primary human CD1c^+^ DCs.

Synthetic abortive HIV-1 Cap-RNA_58_ contains a 5′cap and a secondary TAR loop structure and is a ligand for DDX3, which leads to the induction of type I IFN responses (27). The HIV-1 Cap-RNA_58_-induced type I IFN responses were similar if not stronger than those observed by poly-I:C, which triggers RIG-I and MDA5. RIG-I and MDA5 distinguish the recognition of their viral ligands based on RNA structure and length. RIG-I recognizes ssRNAs and short dsRNAs, whereas MDA5 recognizes longer dsRNAs (42). Soto-Rifo et al. have described that DDX3 recognizes viral RNA constructs due to the presence of a 5′cap in close proximity to a complex secondary structure (30). Whether DDX3 is able to distinguish between different viral RNA lengths and adapts subsequent immune activation is unknown. Here we aimed to assess whether differences in HIV-1 Cap-RNA construct length would lead to varying immune activation levels. We observed that the length of the synthetic viral ligand did not affect the strength of the antiviral type I IFN responses as both HIV-1 Cap-RNA_58_ and HIV-1 Cap-RNA_630_ induced similar levels of *IFNB* and *ISG* mRNA expression. Similar levels of pro-inflammatory cytokines were also observed. Whereas, HIV-1 Cap-TAR_58_ induced type I IFN responses, HIV-1 control RNA did not result in *IFNB* mRNA expression, indicating that the 5′cap is required for recognition by DDX3 and subsequent induction of antiviral responses. Thus, HIV-1 Cap-RNA_58_ induces potent type I IFN in DCs via DDX3 and MAVS-dependent signaling, due to the presence of a 5′cap and complex secondary TAR loop structure.

Besides type I IFN responses, HIV-1 Cap-RNA_58_ resulted in increased upregulation of costimulatory molecules CD80, CD83, and CD86. Furthermore, HIV-1 Cap-RNA_58_ enhanced expression of HLA-DR but not CD40. Our data further indicate that DC maturation is dependent on both type I IFN and NF-κB activation. It remains to be established whether the effect of NF-κB is mediated via IFNβ or that other cytokines activated by NF-κB further affect DC maturation in combination with IFNβ. It has previously been described for various cell lines that DDX3 expression knockdown results in decreased NF-κB p65 phosphorylation and cytokine responses suggesting that DDX3 plays a stimulatory role in NF-κB signaling (43). In addition, Ku et al. (44) described that in THP-1-differentiated macrophages DDX3 is important for TNF, IL-1β, CCL2, and CCL5 expression as knockdown of DDX3 expression impaired cytokine and chemokine expression in response to LPS and poly-I:C stimulations. Besides affecting pro-inflammatory cytokine responses, DDX3 knockdown also led to impaired migration and phagocytic capacities of THP-1-differentiated macrophages (44). Although it is unclear whether these functions can be induced by viral ligands, these data potentially imply that DDX3 could be involved in orchestrating various important functions in DCs. Our data underscore the importance of DDX3 as a viral sensor important for the induction of antiviral immunity.

We observed that HIV-1 Cap-RNA_58_ induced expression of pro-inflammatory cytokines IL-6 and TNF, which are important for both innate and adaptive immune responses. Furthermore, HIV-1 Cap-RNA_58_ induced *IL12A* but not *IL12B* mRNA expression which resulted in the absence of heterodimeric IL-12p70 protein that is crucial for the induction of T_H_1 differentiation. The observed lack of IL-12p70 upon stimulation with HIV-1 Cap-RNA_58_ might explain skewing of T helper differentiation toward a T_H_2 phenotype by HIV-1 Cap-RNA_58_-treated DCs. Furthermore, we observed HIV-1 Cap-RNA_58_-dependent induction of *IL27A*, encoding for one of the subunits of heterodimeric IL-27 protein, important in the induction of follicular T helper (T_FH_) cells (45). Both T_H_2 and T_FH_ cells are important for the induction of antibody responses against invading pathogens including viruses (46). T_FH_ cells are important for the formation and maintenance of germinal centers (GCs) and subsequent differentiation of B cells in GCs (46). Once a B cell exits the GC, T_H_2-induced IL-4 production can direct class switching from immunoglobulin G (IgG) to IgE antibodies (47, 48). Recent studies show that T_FH_ responses are required to induce broadly neutralizing antibodies against HIV-1 (49–51). Although it is unclear yet whether HIV-1 Cap-RNA_58_ induces a T_FH_ phenotype, the cytokine responses and T_H_2 differentiation suggest that HIV-1 Cap-RNA_58_ can be useful in vaccines to induce neutralizing antibodies against HIV-1 or other viruses.

Microbial LPS is increased in serum of HIV-1 infected individuals due to intestinal damage upon CD4^+^ T cell depletion. LPS as a potent immunostimulatory compound could be involved in inflammatory responses during chronic phase of infection (52, 53). Interestingly, several studies suggest that HIV-1 replication in latent infected cells produces short abortive RNAs such as the DDX3 ligand HIV-1 Cap-RNA_58_ (54, 55). Our study suggest that these abortive RNAs can induce inflammatory responses. Thus, besides increased translocation of microbial LPS, the production of HIV-1 Cap-RNA_58_ in latent infected cells can also contribute to immune activation observed in HIV-1 infected individuals during chronic phase of infection.

In conclusion, DDX3 is a highly versatile protein involved in a multitude of cellular processes. During HIV-1 infection, the dual role of DDX3 in exerting both proviral and antiviral capacities provides insight in its complexity and to the various roles DDX3 might play in establishing immunity. Here we have identified the antiviral role of DDX3 upon sensing of a viral-derived RNA and how its ligands can be used as adjuvants. Our data strongly indicate that HIV-1 Cap-RNA_58_ induces potent antiviral innate and adaptive immune responses in human DCs or directed by human DCs. Our data shows that DDX3 is a pattern recognition receptor and its synthetic ligands can be used as adjuvants to induce potent immune responses.

## Data Availability Statement

All datasets generated for this study are included in the article/Supplementary Material.

## Ethics Statement

The studies involving human participants were reviewed and approved by Amsterdam University Medical Centers, location AMC Medical Ethics Committee according to the Declaration of Helsinki. The patients/participants provided their written informed consent to participate in this study.

## Author Contributions

MS designed, performed, and interpreted most experiments and prepared the manuscript. JS helped with study design. JH performed HIV-1 infections and subsequent FACS analyses. TK and EZ-W performed nuclear extract isolations. SG helped with study design and interpretation of data. TG supervised all aspects of this study.

### Conflict of Interest

The authors declare that the research was conducted in the absence of any commercial or financial relationships that could be construed as a potential conflict of interest.
