# Supplementary figures and images for: Synthetic Abortive HIV-1 RNAs Induce Potent Antiviral Immunity
Source: Front Immunol. 2020 Jan 23;11:8. doi: 10.3389/fimmu.2020.00008 (PMC6990453; doi:10.3389/fimmu.2020.00008)

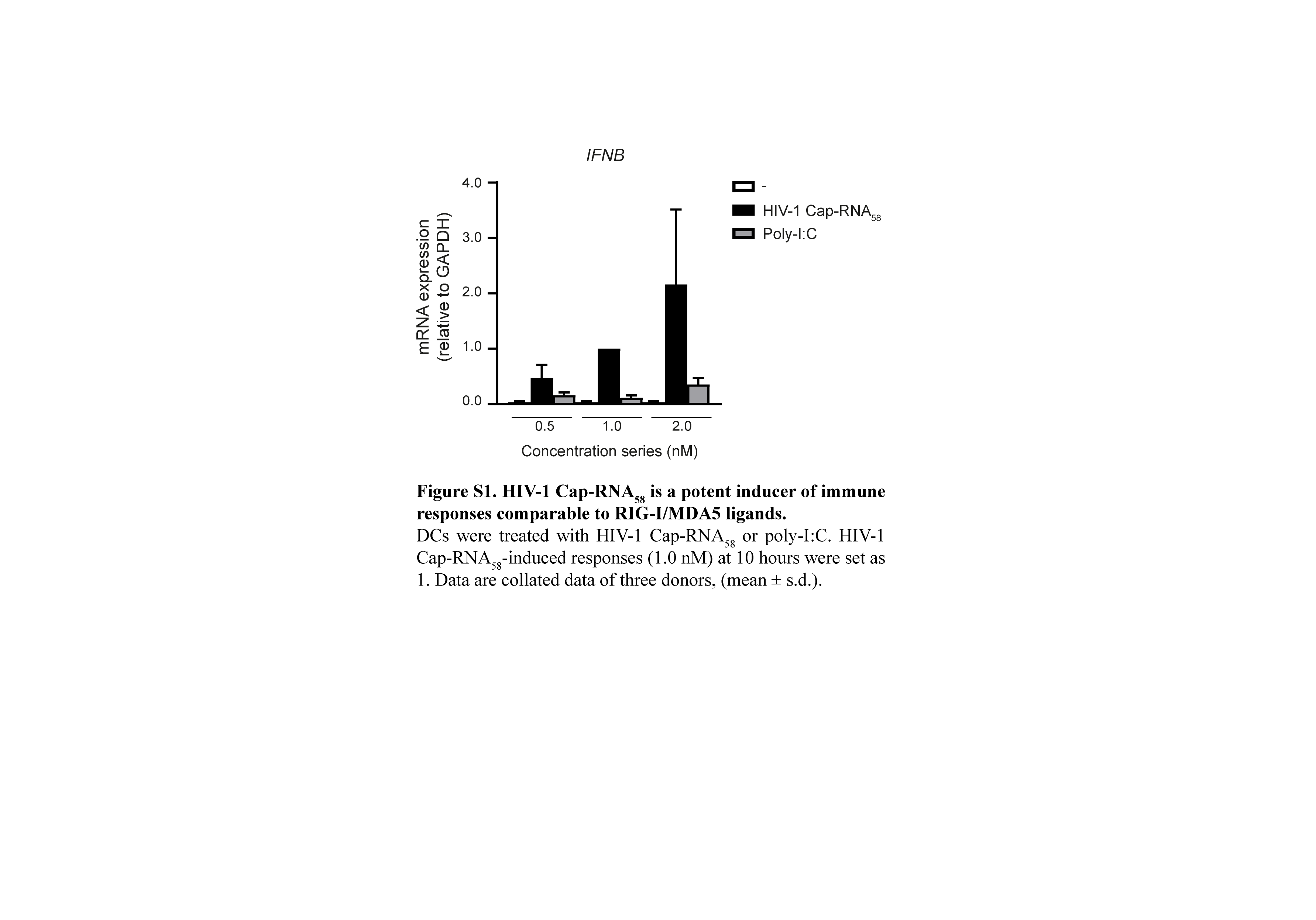

Supplement: Supplementary file 1 [file Image_1.tif]

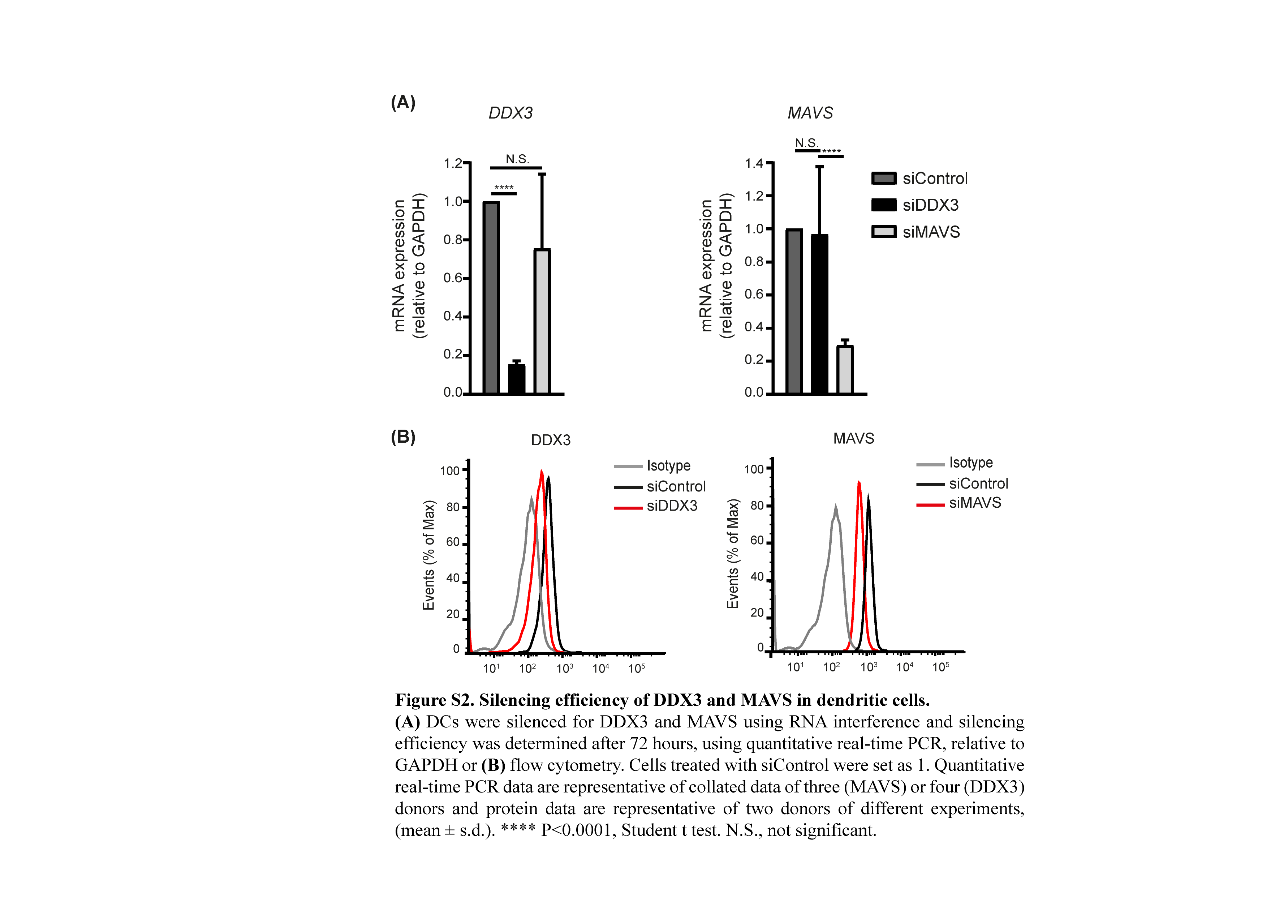

Supplement: Supplementary file 2 [file Image_2.tif]

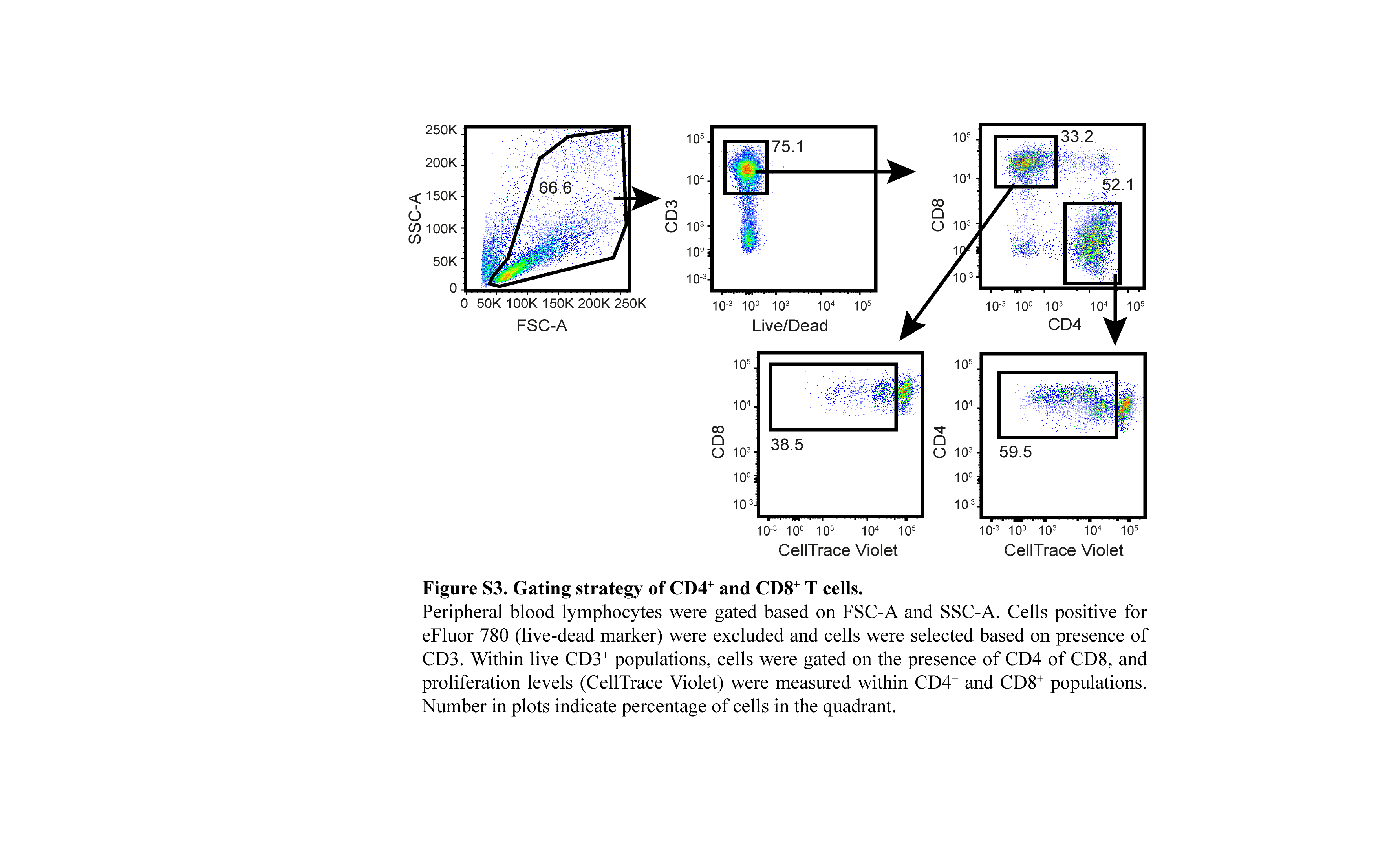

Supplement: Supplementary file 3 [file Image_3.TIF]

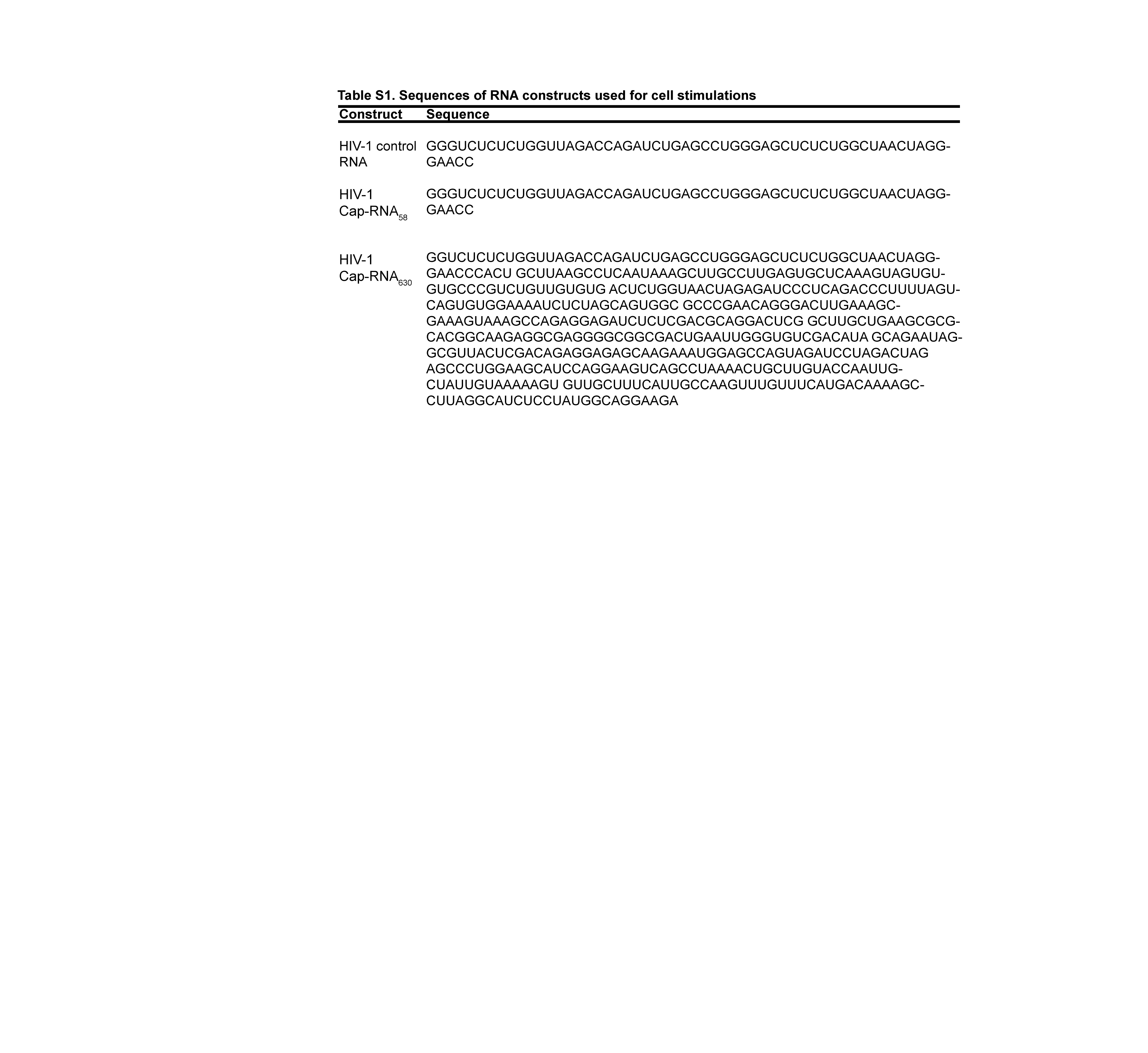

Supplement: Supplementary file 4 [file Table_1.docx]

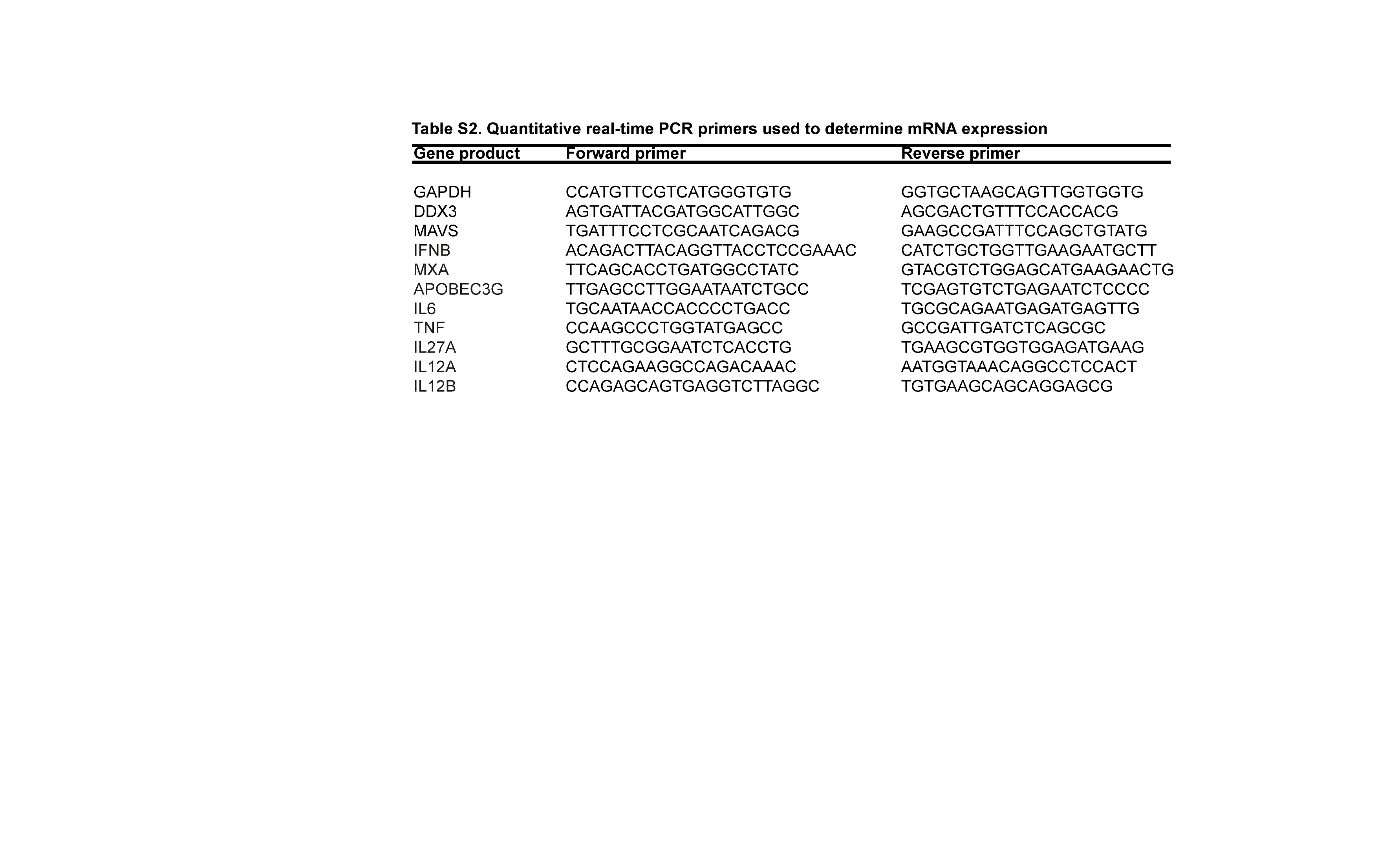

Supplement: Supplementary file 5 [file Table_2.docx]
